# Analysis of the effect and correlation of the co-care model on the diagnosis and treatment of type 2 diabetes patients

**DOI:** 10.1515/med-2026-1386

**Published:** 2026-05-08

**Authors:** Bo An, Fang Chen, HuiXian Yan

**Affiliations:** Beijing Haidian Hospital (Haidian Section of Pecking University Third Hospital), 100080 Beijing, China

**Keywords:** co-care, type 2 diabetes patients, HbA1c target achievement, correlation analysis

## Abstract

**Objectives:**

The traditional diabetes management model focuses primarily on in-hospital treatment, with limited support for patients’ self-management at home. The co-care model, integrating both online and offline components, offers a promising strategy for continuous patient support, but its effectiveness in real-world settings requires further evaluation. This study aimed to evaluate the effectiveness of a one-year co-care model on glycemic control and other cardiometabolic parameters in patients with type 2 diabetes (T2DM), and to explore the association between patient engagement level (categorized by online and offline activity) and health outcomes.

**Methods:**

We conducted an observational study of 1,741 T2DM patients from the Endocrinology Department of Hospital in Beijing enrolled from December 2020 to November 2024. Patients were managed under a co-care model for over one year, involving regular offline follow-ups and an online application for education, monitoring, and communication. Patients were categorized into four groups based on their online and offline activity levels: inactive both, offline-active only, online-active only, and active both. The primary outcome was the proportion achieving the HbA1c target at one year. Secondary outcomes included blood pressure and LDL-C control rates, a composite “3B” target (HbA1c, BP, and LDL-C), and changes in body mass index (BMI). Statistical analyses included descriptive statistics, chi-square/ANOVA tests, and multivariate logistic regression.

**Results:**

After one year of common care mode management, patients with type 2 diabetes had lower glycosylated hemoglobin, higher glycosylated standard rate and lower low-density lipoprotein cholesterol (LDL-C) standard rate, lower glycosylated rate and lower blood pressure, and the proportion of 18–28 kg/m^2^ in BMI segment would increase. In addition, through the management of common care mode, the rate of poor glycated hemoglobin (HbA1c), systolic blood pressure (SBP), diastolic blood pressure (DBP), rate of poor blood pressure and LDL-C of type 2 diabetes patients have improved.

**Conclusions:**

The co-care model effectively improves cardiometabolic outcomes of blood glucose control in patients with type 2 diabetes. The co-care model was associated with significant improvements in glycemic control and other cardiometabolic parameters among T2DM patients over one year. Higher patient engagement, particularly through combined online and offline activities, was strongly linked to better glycemic outcomes. These findings support the clinical value and potential for broader implementation of the integrated co-care model in diabetes management.

## Introduction

Type 2 diabetes (T2DM) is a common metabolic disease characterized by insulin resistance and relative insulin secretion deficiency. In recent years, research on the diagnosis and treatment effect of type 2 diabetes patients has gradually increased, covering drug treatment, lifestyle intervention, monitoring and complication management [1]. Among them, drug therapy mainly includes classic drugs (such as metformin as the first-line treatment for T2DM, which has good effects in controlling blood sugar, weight loss, and cardiovascular protection), new drugs (such as GLP-1 receptor agonists (such as liraglutide) and SGLT2 inhibitors (such as dapagliflozin), which not only effectively reduce blood sugar, but also have weight loss and cardiovascular protection effects), and combination therapy (the combination of hypoglycemic drugs with different mechanisms can more effectively control blood sugar levels) [2], [3], [4]. In terms of lifestyle interventions, dietary management (a reasonable diet structure can significantly improve blood sugar control) and exercise (regular physical activity has been shown to improve insulin sensitivity and lower blood sugar levels) are the main research directions; In addition, it also includes self-management and education (improving patients’ awareness and self-management abilities of the disease), as well as complication management (such as prevention and treatment of cardiovascular complications in T2DM patients), etc. [5].

The management of diabetes patients in China has always adopted the traditional medical model, that is, diabetes patients go offline to see the hospital, and then go home for self-management outside the hospital after the diagnosis and treatment. In fact, the hospital visit time of patients only accounts for about 4 % every year, and the home self-blood glucose management time accounts for about 96 %. At present, the management of diabetes in China mainly focuses on the treatment of diabetes patients, while ignoring the home self-blood glucose management. Because most of diabetes patients lack professional guidance and daily management, the level of self-management behavior during home is low, and it is difficult to adjust their living habits, diet composition, etc. according to their own conditions, which requires timely communication with medical staff to have a correct understanding of the evolution of complex diseases and adjust management goals and self-management plans to adapt to the changes in the condition [6]^.^ At present, the traditional diagnosis and treatment model of diabetes patients obviously lacks the part of out of hospital management, which is difficult to support frequent communication between doctors and patients.

With the diversified development of diagnosis and treatment methods for type 2 diabetes in recent years, the co-care model is also increasingly valued in the management of type 2 diabetes patients. This model emphasizes the cooperation of medical teams, including doctors, nurses, nutritionists, mental health experts, etc., in order to provide comprehensive patient care. This model not only focuses on the physical health of patients, but also pays attention to psychological and social factors, thereby improving the quality of life and treatment effectiveness of patients [7]. Its characteristics include [8], [9], [10]: (1) multidisciplinary team: involving endocrinologists, nutritionists, health managers, psychological counselors, etc., forming a collaborative working mechanism; (2) Individualized treatment plan: Develop a personalized treatment plan based on the patient’s specific situation, taking into account factors such as lifestyle and psychological state; (3) Patient participation: Encourage patients to participate in their own treatment process, enhance their initiative and sense of responsibility in health management; (4) Regular evaluation and feedback: By regularly monitoring and evaluating the health status of patients, timely adjustments to treatment plans can be made. The co-care model provides a new management strategy for patients with type 2 diabetes. Through the cooperation of multidisciplinary teams and the active participation of patients, it is expected to improve the treatment effect and quality of life of patients to a certain extent. In order to explore the effect of type 2 diabetes patients in terms of blood sugar control (blood sugar compliance rate, poor blood sugar rate) under the common care mode, we selected type 2 diabetes patients who visited the Endocrine Department of Hospital in Beijing as the research objects. Through in hospital treatment and out of hospital common care mode, we managed the patients continuously, and monitored and evaluated the blood sugar index, low density lipoprotein cholesterol, body mass index and other indicators of patients after 1 year of common care mode management.

## Object and method

### Research object

Type 2 diabetes patients who visited the Endocrinology Department of Hospital in Beijing from December 2020 to November 2024, and joined the co-care for more than 1 year, with no missing data on age, course of disease, baseline and glycated hemoglobin (HbA1c) after 1 year, were selected as the study subjects. The number of patients who met the above conditions was 1,741.

### Research group

(1) Offline management indicators (Indicators for in-hospital management): The offline management indicator is the number of follow-up visits to the hospital. Patients who have visited the hospital three or more times in a year are defined as offline active patients, otherwise they are defined as offline inactive patients.
(2) Online management indicators (Indicators for out-hospital management): Co-care model is carried out through regular follow-up to patients with type 2 diabetes, pushing relevant diagnosis and treatment measures and precautions though application APP. Among them, the main indicators for online management include the total time spent logging into the app, the total number of times the app is logged in, the total number of times blood sugar is monitored, the number of times food photos are uploaded, the number of times articles are read, the number of times videos are watched on the app, the number of times hypoglycemia occurs, the number of messages sent to caregivers through the app, and the duration of communication with caregivers through the app. These indicators are divided into three segments based on the median (0 for 0 times, 1 for times less than or equal to the median, and 2 for times greater than the median). The three segments of all indicators are added together to form a value, and the median is used to distinguish whether they are active online.

According to the level of online and offline activity, it can be divided into four groups: inactive both online and offline, active only offline, active only online, and active both online and offline.

### Main indicators and evaluation criteria

The height, weight, blood pressure, confirmed complications, discharge diagnosis, blood lipid test results (including HbA1c), low-density lipoprotein cholesterol (LDL-C) and other information of the enrolled patients with type 2 diabetes were collected. The detection instruments and equipment for the above indicators were in line with the national indoor quality control standards, and the results were true and effective.

Evaluation criteria: Glycated hemoglobin (HbA1c) meets the standard: when the age is <65 years old, HbA1c <7 %; When the age is ≥65 years old, HbA1c <7.5 %. HbA1c deficiency: HbA1c control is considered poor when HbA1c >9 %. Low density lipoprotein cholesterol (LDL-C) meets the standard: LDL-C <2.6 mmol/L. Blood pressure meets the standard: blood pressure <130/80 mmHg. Blood pressure not up to standard: blood pressure >140/90 mmHg. 3B comprehensive standard: Simultaneously meeting the standards for glycated hemoglobin, blood pressure, and blood lipids.

The primary outcome is explicitly defined as: the proportion of patients achieving the HbA1c target (HbA1c <7.0 % for age <65 years; HbA1c <7.5 % for age ≥65 years) at the one-year follow-up. The secondary outcomes are explicitly listed as: (1) the proportion of patients achieving the blood pressure target (<130/80 mmHg); (2) the proportion achieving the LDL-C target (<2.6 mmol/L); (3) the proportion achieving the “3B” composite target (simultaneous control of HbA1c, BP, and LDL-C); and (4) the proportion with poor control (HbA1c >9.0 % or BP >140/90 mmHg).

### Statistical analysis

Use R.4.2.1 software for statistical analysis of data. Statistical description of data [11]: Quantitative data is described using mean ± standard deviation (requiring the population to follow a normal distribution) or median to interquartile range [M(P25, P75), used to describe the concentration and dispersion trends of asymmetric distributions, it is a concept in the percentile that sorts a set of data from small to large, corresponding to the value at the x% position.], not requiring the population to follow a normal distribution; Qualitative data is described using frequency (percentage). Quantitative data is analyzed using analysis of variance (which requires the population to follow a normal distribution and have homogeneous variance) or Kruskal Wallis *H* test; Qualitative data is analyzed using a chi-square test (if 1≤ theoretical frequency <5, a corrected chi-square value needs to be calculated). Using logistic regression analysis [12] to explore the factors that affect the achievement of glycation standards.

### Ethical considerations

That written informed consent was obtained from all participating patients prior to their enrollment. The consent form clearly explained the study’s purpose, procedures, potential risks and benefits, and the right to withdraw at any time without affecting their standard medical care.

All data were anonymized and de-identified immediately after collection. Patient identifiers (names, ID numbers, contact details) were replaced with unique study codes, and the code-key was stored separately on a password-protected, secure hospital server with strict access controls limited to the principal investigators.

All study procedures were conducted in accordance with the principles outlined in the Declaration of Helsinki.

## Results and analysis

### General clinical information of patients

Statistical methods were used to describe the basic characteristics of patients with type 2 diabetes, including the age of initial diagnosis, course of initial diagnosis, age segmentation, disease segmentation, gender and various diagnosis and treatment indicators. 1,741 patients were divided into four groups based on their level of activity: n=296 patients were inactive both online and offline, n=417 patients were active only offline, n=329 patients were active only online, and n=599 patients were active both online and offline.

According to the description of baseline data in Table 1, combined with the analysis of characteristics of diabetes patients with different activeness (online and offline), the results show that there are significant differences in multiple indicators among the four groups. The age of initial diagnosis varies significantly among different groups, with an overall age of 50.91 ± 12.87 years. The group with inactive online and offline activities had the highest age of initial diagnosis (53.05 ± 12.89 years), while the group with active online activities had the lowest age of initial diagnosis (48.68 ± 13.44 years). In terms of disease duration, the overall initial diagnosis duration of patients was 5.99 ± 7.27 years. The online active group had the shortest disease duration (3.70 ± 5.57 years), while the offline inactive group had the longest disease duration (8.20 ± 7.94 years). The baseline average level of glycated hemoglobin was 9.09 ± 2.29 %, with significant differences between different groups. The baseline HbA1c target achievement rate was highest in the offline active group (25.95 %), while the baseline glycation failure rate was highest in the online active group (52.78 %). There were significant differences in diastolic blood pressure (DBP) among the groups, with an overall value of 83.99 ± 10.66 mmHg. The baseline DBP was highest in the active group both online and offline (85.32 ± 10.56 mmHg). The baseline average value of LDL-C was 3.06 ± 1.05 mmol/L, with the lowest LDL-C level observed in the offline active group (2.87 ± 1.04 mmol/L), and the highest LDL-C compliance rate observed in this group (44.32 %). The overall BMI is 26.74 ± 4.43 kg/m^2^, with little difference between groups. BMI is mainly concentrated in the range of 24–28 kg/m^2^.

**Table 1:** Description of baseline data of patients with type 2 diabetes.

Variable quantity	Total, n=1,741	Online and offline activity level				
		Not active both online and offline, n=396	Purely offline active, n=417	Purely online active, n=329	Active both online and offline, n=599	p-Value
Age of initial diagnosis	50.91 ± 12.87	53.05 ± 12.89	52.49 ± 12.00	48.68 ± 13.44	49.63 ± 12.80	<0.001
Initial diagnosis course	5.99 ± 7.27	8.20 ± 7.94	8.34 ± 8.08	3.70 ± 5.57	4.16 ± 6.04	<0.001
Age segmentation						<0.001
<45	582 (33.47 %)	114 (28.86 %)	110 (26.38 %)	131 (39.94 %)	227 (37.90 %)	
45–60	887 (51.01 %)	201 (50.89 %)	246 (58.99 %)	152 (46.34 %)	288 (48.08 %)	
60–75	234 (13.46 %)	66 (16.71 %)	54 (12.95 %)	38 (11.59 %)	76 (12.69 %)	
>75	36 (2.07 %)	14 (3.54 %)	7 (1.68 %)	7 (2.13 %)	8 (1.34 %)	
Segmented disease course						<0.001
<2 year	755 (43.95 %)	115 (29.04 %)	116 (28.57 %)	191 (58.23 %)	333 (56.63 %)	
2–5 year	268 (15.60 %)	66 (16.67 %)	63 (15.52 %)	53 (16.16 %)	86 (14.63 %)	
5–10 year	229 (13.33 %)	61 (15.40 %)	70 (17.24 %)	36 (10.98 %)	62 (10.54 %)	
10–15 year	227 (13.21 %)	71 (17.93 %)	73 (17.98 %)	25 (7.62 %)	58 (9.86 %)	
>15 year	239 (13.91 %)	83 (20.96 %)	84 (20.69 %)	23 (7.01 %)	49 (8.33 %)	
Gender						0.400
Female	646 (37.11 %)	156 (39.39 %)	158 (37.89 %)	126 (38.30 %)	206 (34.39 %)	
Male	1,095 (62.89 %)	240 (60.61 %)	259 (62.11 %)	203 (61.70 %)	393 (65.61 %)	
Glycosylated hemoglobin, %	9.09 ± 2.29	8.96 ± 2.26	8.86 ± 2.27	9.39 ± 2.25	9.16 ± 2.32	0.006
HbA1c compliance rate, %	373 (22.15 %)	92 (23.96 %)	102 (25.95 %)	53 (16.36 %)	126 (21.61 %)	0.015
Hypoglycation rate, %	794 (47.15 %)	164 (42.71 %)	176 (44.78 %)	171 (52.78 %)	283 (48.54 %)	0.037
SBP, mmHg	133.50 ± 16.86	132.67 ± 16.59	132.49 ± 16.83	133.98 ± 17.75	134.51 ± 16.52	0.200
DBP, mmHg	83.99 ± 10.66	82.32 ± 9.52	83.45 ± 10.66	84.29 ± 11.80	85.32 ± 10.56	<0.001
Blood pressure compliance rate, %	394 (22.67 %)	95 (24.05 %)	92 (22.06 %)	82 (24.92 %)	125 (20.94 %)	0.5
Poor blood pressure, %	345 (19.85 %)	61 (15.44 %)	81 (19.42 %)	74 (22.49 %)	129 (21.61 %)	0.4
LDL-C, mmol/L	3.06 ± 1.05	3.03 ± 1.17	2.87 ± 1.04	3.25 ± 0.99	3.11 ± 0.98	<0.001
LDL-C compliance rate, %	542 (34.83 %)	132 (37.50 %)	160 (44.32 %)	74 (25.34 %)	176 (31.94 %)	<0.001
Composite 3B target rate, %	41 (2.67 %)	12 (3.43 %)	6 (1.69 %)	6 (2.06 %)	17 (3.14 %)	0.4
BMI, kg/m^2^	26.74 ± 4.43	26.48 ± 4.04	26.78 ± 4.43	27.07 ± 4.94	26.70 ± 4.38	0.800
BMI segmentation						
<18, kg/m^2^	21 (1.21 %)	4 (1.01 %)	8 (1.92 %)	3 (0.91 %)	6 (1.01 %)	
18–24, kg/m^2^	437 (25.14 %)	106 (26.84 %)	92 (22.06 %)	86 (26.14 %)	153 (25.63 %)	
24–28, kg/m^2^	692 (39.82 %)	163 (41.27 %)	184 (44.12 %)	121 (36.78 %)	224 (37.52 %)	
>28, kg/m^2^	588 (33.83 %)	122 (30.89 %)	133 (31.89 %)	119 (36.17 %)	214 (35.85 %)	

Overall, the gender of patients in different groups of online and offline activity levels BMI, There was no significant difference in baseline levels of blood pressure compliance rate and composite 3B target rate. There are significant differences in age, disease duration, glycation level, HbA1c target achievement rate, glycation failure rate, and LDL-C baseline level. Specifically, patients in the online active group are generally younger and have a shorter course of illness, while those in the offline active group are older and have a longer course of illness. Online active patients have higher baseline levels of glycated hemoglobin, while offline active patients have lower baseline levels. Among them, the pure offline active glycation had the highest compliance rate (25.95 %), the lowest LDL-C (2.87 ± 1.04 mmol/L), and the highest LDL-C compliance rate (44.32 %). Regarding the offline management indicator (number of hospital visits), the mean (SD) follow-up visits per year were: 1.2 (0.8) in the “inactive both online and offline” group, 4.5 (1.3) in the “active only offline” group, 1.5 (0.9) in the “active only online” group, and 4.3 (1.5) in the “active both online and offline” group. These figures validate our grouping criteria based on the threshold of ≥3 visits per year for offline activity.

### Comparison of co-care model management before and after 1 year

Table 2 shows the comparative data before and after 1 year of management under the shared care model, and the feature data is subdivided according to online and offline activity levels. Furthermore, the differences in changes between the groups were further refined and compared, and the comparative analysis results are shown in Table 3. The analysis results showed that after 1 year of management under the co-care model, there were significant differences in multiple indicators between the four groups and the baseline.

**Table 2:** Comparison data of management before and after 1 year under the co care mode.

Index	Baseline level	Management level for 1 year	p-Value
Glycosylated hemoglobin, %	9.09 ± 2.29	6.77 ± 1.20	<0.001
HbA1c compliance rate, %	373 (22.15 %)	882 (69.56 %)	<0.001
Hypoglycation rate, %	794 (47.15 %)	74 (5.84 %)	<0.001
SBP, mmHg	133.50 ± 16.86	129.15 ± 11.20	<0.001
DBP, mmHg	83.99 ± 10.66	79.14 ± 8.14	<0.001
Compliance rates of discharge of blood pressure, %	394 (22.67 %)	592 (43.21 %)	<0.001
Hypoglycation rate, %	345 (19.85 %)	68 (4.96 %)	<0.001
LDL-C, mmol/L	3.06 ± 1.05	2.58 ± 0.90	<0.001
LDL-C compliance rate, %	542 (34.83 %)	413 (55.59 %)	<0.001
composite 3B target rate, %	41 (2.67 %)	128 (17.68 %)	<0.001
BMI, kg/m^2^	26.74 ± 4.43	26.22 ± 4.32	<0.001
BMI segmentation			<0.001
<18, kg/m^2^	21 (1.21 %)	14 (1.02 %)	
18–24, kg/m^2^	437 (25.14 %)	399 (29.12 %)	
24–28, kg/m^2^	692 (39.82 %)	560 (40.88 %)	
>28, kg/m^2^	588 (33.83 %)	397 (28.98 %)	

**Table 3:** Characteristic data before and after 1 year of management under the co-care model.

Characteristic indicators	Baseline				After 1 year of management			
	Not active both online and offline, n=396	Purely offline active, n=417	Purely online active, n=329	Active both online and offline, n=599	Not active both online and offline, n=396	Purely offline active, n=417	Purely online active, n=329	Active both online and offline, n=599
BMI	26.48 ± 4.04	26.78 ± 4.43	27.07 ± 4.94	26.70 ± 4.38	26.59 ± 4.30	26.52 ± 4.32	26.28 ± 4.78	25.88 ± 4.19
BMI segmentation								
<18, kg/m^2^	4 (1.01 %)	8 (1.92 %)	3 (0.91 %)	6 (1.01 %)	2 (1.00 %)	3 (0.72 %)	0 (0.00 %)	9 (1.50 %)
18–24, kg/m^2^	106 (26.84 %)	92 (22.06 %)	86 (26.14 %)	153 (25.63 %)	48 (24.00 %)	107 (25.66 %)	52 (33.77 %)	192 (32.05 %)
24–28, kg/m^2^	163 (41.27 %)	184 (44.12 %)	121 (36.78 %)	224 (37.52 %)	92 (46.00 %)	176 (42.21 %)	57 (37.01 %)	235 (39.23 %)
>28, kg/m^2^	122 (30.89 %)	133 (31.89 %)	119 (36.17 %)	214 (35.85 %)	58 (29.00 %)	131 (31.41 %)	45 (29.22 %)	163 (27.21 %)
HbA1c, %	8.96 ± 2.26	8.86 ± 2.27	9.39 ± 2.25	9.16 ± 2.32	7.39 ± 1.42	6.98 ± 1.29	6.72 ± 1.15	6.45 ± 0.95
HbA1c compliance rate	92 (23.96 %)	102 (25.95 %)	53 (16.36 %)	126 (21.61 %)	97 (48.60 %)	243 (63.95 %)	100 (70.92 %)	452 (79.58 %)
Hypoglycation rate	164 (42.71 %)	176 (44.78 %)	171 (52.78 %)	283 (48.54 %)	18 (10.06 %)	35 (9.21 %)	10 (7.09 %)	11 (1.94 %)
SBP	132.67 ± 16.59	132.49 ± 16.83	133.98 ± 17.75	134.51 ± 16.52	129.8 ± 10.46	129.95 ± 11.77	129.38 ± 10.60	128.31 ± 11.15
DBP	82.32 ± 9.52	83.45 ± 10.66	84.29 ± 11.80	85.32 ± 10.56	79.72 ± 7.71	78.65 ± 8.14	77.99 ± 8.45	79.57 ± 8.17
Compliance rates of discharge of blood pressure	95 (24.05 %)	92 (22.06 %)	82 (24.92 %)	125 (20.94 %)	80 (40.00 %)	172 (41.25 %)	77 (50.00 %)	263 (43.91 %)
Poor blood pressure rate	61 (15.44 %)	81 (19.42 %)	74 (22.49 %)	129 (21.61 %)	8 (4.00 %)	14 (3.36 %)	10 (6.49 %)	36 (6.01 %)
LDL-C, mmol/L	3.03 ± 1.17	2.87 ± 1.04	3.25 ± 0.99	3.11 ± 0.98	2.66 ± 0.96	2.52 ± 0.94	2.66 ± 0.90	2.58 ± 0.85
Composite 3B target rate	12 (3.43 %)	6 (1.69 %)	6 (2.06 %)	17 (3.14 %)	7 (7.45 %)	34 (16.11 %)	13 (20.63 %)	74 (20.79 %)

The above p values are all <0.01; HbA1c, glycated hemoglobin.

After 1 year of common care mode management, compared with the baseline, the glycosylated hemoglobin, the rate of reaching the standard of glycosylation, and the rate of poor glycosylation in all groups of type 2 diabetes patients significantly improved from the baseline 9.09 ± 2.29 to 6.77 ± 1.20, the rate of reaching the standard of glycosylation increased from 22.15 % to 69.56 %, and the rate of poor glycosylation decreased from 47.15 % to 5.84 % (p<0.001). And among the groups, the trend showed that the group with both online and offline activity was better than the group with only online activity, better than the group with only offline activity, and better than the group with both online and offline inactivity. Among them, the active group both online and offline had the largest decrease in glycation rate and the lowest value (from 9.16 ± 2.32 % to 6.45 ± 0.95 %), the largest increase in HbA1c target achievement rate and the highest compliance rate (from 21.61 % to 79.58 %), and the largest decrease in glycation failure rate and the lowest value (from 21.61 % to 1.94 %). On the contrary, the active group showed the smallest decrease in glycation both online and offline (from 8.96 ± 2.26 % to 7.39 ± 1.42 %), the smallest increase in HbA1c target achievement rate (from 23.96 % to 48.60 %), and the smallest decrease in glycation failure rate (from 42.71 % to 10.06 %).

After adopting a shared care model for 1 year, blood pressure and lipid control in all groups significantly improved. The overall systolic blood pressure (SBP) decreased from 133.50 ± 16.86 mmHg to 129.15 ± 11.20 mmHg, and the diastolic blood pressure (DBP) decreased from 83.99 ± 10.66 mmHg to 79.14 ± 8.14 mmHg. The blood pressure compliance rate increased from 22.67 % to 43.21 %, and the incidence of poor blood pressure decreased from 19.85 % to 4.96 % (p<0.001). The overall low-density lipoprotein cholesterol (LDL-C) decreased from 3.06 ± 1.05 to 2.58 ± 0.90, and the LDL-C compliance rate increased from 34.83% to 55.59 % (p<0.001). However, there was no significant statistical difference in the degree of improvement of blood pressure and blood lipids among the groups.

In terms of the composite 3B target rate, after 1 year of using the shared care model for management, the overall rate increased from 2.67 % to 17.68 % (p<0.001). The group with both online and offline activity had the highest score (20.79 %), similar to the group with only online activity (20.63 %). The group with only offline activity was in the middle (16.11 %), while the group with neither online nor offline activity had the lowest score, with a compliance rate of only 7.45 %.

In terms of body mass index (BMI), the overall BMI decreased from 26.74 ± 4.43 to 26.22 ± 4.32 (p<0.001) after 1 year of management using a shared care model. In BMI segmentation,> The proportion of 28 kg/m^2^ (obese) decreased by 4.85 % (p<0.001), the proportion of 24–28 kg/m^2^ (overweight) increased by 1.06 %, and the proportion of 18–24 kg/m^2^ (standard body weight) increased by 3.98 %< The proportion of 18 kg/m^2^ (low body weight) decreased by 0.19 %. The specific grouping comparison results showed that the proportion of obese individuals decreased by 1.89 % in the inactive group both online and offline, the proportion of overweight individuals increased by 4.73 %, and the proportion of normal weight decreased by 2.84 %. The proportion of obese individuals in the offline active group decreased by 0.48 %, the proportion of overweight individuals decreased by 1.91 %, and the proportion of normal weight individuals increased by 3.6 %. The obesity rate of the online active group decreased by 6.95 %, the overweight rate increased by 0.23 %, and the normal weight rate increased the most significantly by 7.63 %. The proportion of obese individuals in both online and offline active groups decreased the most by 8.73 %, the proportion of overweight individuals increased by 1.98 %, and the proportion of normal weight individuals increased by 6.42 %.

Overall, after 1 year of common care mode management, patients with type 2 diabetes have lower glycosylated hemoglobin, higher glycosylated standard rate, lower rate of poor glycosylation, higher rate of blood pressure and LDL-C standard rate, and higher rate of 3B standard rate. The proportion of 18–28 kg/m^2^ in BMI segment will increase, and the changes of these indicators are considered positive for patients with type 2 diabetes [13], 14].

### Analysis of influencing factors

Multivariate logistic regression analysis was performed to identify factors independently associated with achieving the HbA1c target. The model was adjusted for the following potential confounders: age category, disease duration category, baseline BMI, and activity level group. The results of both univariate and multivariate analyses are presented in Table 4.

**Table 4:** Analysis of factors influencing the compliance rate of glycated hemoglobin (HbA1c).

Influence factor	Univariate analysis (OR (95CI, p)	Multivariate analysis (OR (95CI, p)
Age segmentation (<45 years old as a covariate)		
45–60 years old	0.73 (0.56–0.95, p=0.018)	1.15 (0.84–1.58, p=0.380)
60–75 years old	0.94 (0.64–1.40, p=0.767)	1.96 (1.22–3.17, p=0.006)
>75 years old	0.39 (0.16–0.92, p=0.032)	0.99 (0.36–2.68, p=0.980)
Disease course segmentation (with <2 years as a sub variable)		
2–5 years old	0.57 (0.39–0.83, p=0.004)	0.61 (0.41–0.92, p=0.018)
5–10 years old	0.25 (0.17–0.36, p<0.001)	0.27 (0.18–0.40, p<0.001)
10–15 years old	0.23 (0.16–0.33, p<0.001)	0.26 (0.17–0.39, p<0.001)
>15 years old	0.17 (0.12–0.25, p<0.001)	0.19 (0.12–0.30, p<0.001)
Whether blood pressure meets the standard or not		
Reach the standard	0.89 (0.67–1.17, p=0.404)	
LDL-C compliance rate		
Reach the standard	0.85 (0.67–1.09, p=0.202)	
BMI	1.05 (1.02–1.08, p<0.001)	1.04 (1.01–1.07, p=0.018)
Activity level (variable based on inactivity both online and offline)		
Purely offline active	2.05 (1.45–2.91, p<0.001)	2.38 (1.62–3.50, p<0.001)
Purely online active	2.38 (1.57–3.60, p<0.001)	2.09 (1.32–3.32, p=0.002)
Active both online and offline	4.11 (2.93–5.77, p<0.001)	3.67 (2.52–5.35, p<0.001)

In this multiple factor analysis, assuming all other variables remain constant, the 60–75 age group had the highest compliance rate 1.96 times that of the <45 age group (p=0.06), while the remaining groups showed no significant correlation with the <45 age group in terms of HbA1c target achievement rate.

In terms of disease course, whether in univariate analysis or multivariate analysis, as the disease course prolongs, the HbA1c target achievement rate of patients gradually decreases. In multivariate analysis, the HbA1c target achievement rate of the 2–5 year group was 0.61 times that of the <2-year group (p=0.018), while the HbA1c target achievement rates of the 5–10 year and 10–15 year groups were 0.27 times (p<0.001) and 0.26 times (p<0.001), respectively, of the <2-year group The HbA1c target achievement rate of the 15 year group is only 0.19 times that of the <2-year group (<0.001).

There is no significant correlation between blood pressure compliance, LDL-C compliance, and HbA1c target achievement rate. The change in BMI has a positive impact on the HbA1c target achievement rate, with an OR of 1.05 (p<0.001) in univariate analysis and 1.04 (p=0.018) in multivariate analysis.

The activity level has the most significant impact on the compliance rate of glycated hemoglobin (HbA1c). Both online and offline activities can significantly improve the HbA1c target achievement rate, and the OR values of the two groups are similar. In multivariate analysis, the OR value for the offline active group was 2.38 (p<0.001), while the OR value for the online active group was 2.09 (p=0.002). If both online and offline activities are active, it has a greater impact on the glycation achievement rate. When other variables remain constant, the glycation achievement rate of the group with both online and offline activities is 3.67 times higher than that of the group without both activities (p<0.001), indicating that patient activity has a significant positive impact on the glycation achievement rate [15].

## Discussions

The population of diabetes in China has continued to expand in the past 30 years. The epidemiological survey of diabetes shows that the prevalence of diabetes in China by 2018–2019 will reach 11.9 % according to the WHO standard in 1999 and 12.4 % according to the ADA standard in 2010 [16]. Although the population of diabetes is still dominated by middle-aged and elderly patients, with the reduction of physical activity, sedentary, unhealthy eating habits of contemporary young people, and the increasingly prominent problems of overweight and obesity, the onset age of diabetes is becoming younger and younger. Research shows that the median age of diabetes patients in China was 55.8 years in 2013 and 51.3 years in 2018. Not only in China, the younger trend of type 2 diabetes is also becoming a serious challenge worldwide. As the population of diabetes patients continues to expand and become younger, the management concept of diabetes has gradually changed [17]. The co-care model is a new medical model to manage and treat diabetes, which has been promoted in China in recent years. Originating from the UK, it is a patient-centered team care model. The care team includes primary care physicians, specialist physicians, nursing staff, nutritionists, pharmacists, clinical psychologists, and social workers.

Our hospital started to establish a shared care outpatient clinic in December 2020, and it has been more than 4 years since then. As of December 2024, a total of 3,512 patients have been managed. By analyzing the basic information of patients in different groups, we found that elderly patients with longer disease duration tend to prefer traditional offline management and have lower activity levels. This may be related to factors such as elderly patients preferring to follow traditional offline medical treatment methods, lack of proficiency in mobile applications, and inconvenient mobility [18], 19]. Compared to other groups, the overall age of the online active group is younger and the duration of illness is shorter, especially in the pure online active group where the age is the youngest and the duration of illness is the shortest. This suggests that young patients are more inclined towards convenient online management mode rather than offline medical treatment, which is also more in line with modern people’s demand for fast pace and convenience.

Baseline data shows that before being included in management, the offline active group, although older overall and with longer disease duration, had the best glycation values, HbA1c target achievement rate, glycation failure rate, LDL-C, and LDL-C compliance rate. This may be due to the fact that this group of people has a longer disease course (about 8 years) and started medication treatment before enrollment. However, in the online active group, the overall age is relatively small, the course of disease is relatively short (about 4 years), there are many patients with diabetes or early diabetes, and drug treatment has not been carried out or is not sufficient.

The superior glycemic outcomes observed in the “active both online and offline” group could partially be attributed to their younger age and shorter disease duration at baseline. However, after adjusting for these and other covariates in the multivariate analysis, higher activity levels remained significantly and independently associated with a greater likelihood of achieving HbA1c control (Table 4). This strengthens the argument that active engagement in the co-care model itself contributes positively to glycemic management.

Compared to baseline data, the overall outcomes after 1 year of co-care management showed significant improvements across all measures, including glycemic control (HbA1c), blood pressure, blood lipids, body weight, and the composite 3Bcompliance (target achievement rate for blood glucose, blood pressure, and lipids). Notably, a detailed subgroup analysis revealed that every group, regardless of their activity level, exhibited significant improvements in all indicators over the one-year period – including the group with the lowest activity level (inactive both online and offline). This suggests that participation in the co-care model, irrespective of the degree of engagement, can lead to positive and sustained impacts on key health indicators such as blood glucose, lipids, blood pressure, and body weight. These findings underscore the intrinsic clinical value of the co-care model and provide robust evidence-based support for its broader implementation in diabetes management.

However, the patient-centered management model of shared care still has an inseparable relationship between management effectiveness and the level of patient participation [20]. Data analysis shows that the activity level of patients is particularly closely related to the improvement of glycation related indicators (glycation values, compliance rates, and failure rates). The higher the activity level, the more significant the improvement in glycation related data. The overall performance shows that the online and offline active group is better than the pure online active group, better than the pure offline active group, and better than the online and offline inactive group. The analysis of the influencing factors of HbA1c target achievement rate shows that, with other variables unchanged, the HbA1c target achievement rate of the group with both online and offline activity is 3.67 times higher than that of the group without both online and offline activity, indicating the significant impact of activity on blood glucose management effectiveness. At the same time, influenced by glycation standards, the composite 3B target rate of the high activity group is also higher, especially the group with high online activity (both online and offline active, purely online active) has the highest composite 3B target rate, obviously due to the offline active group or the inactive group. The improvement in patient weight is also similar to the composite 3B target rate, manifested as the online active group (both online and offline active, pure offline active) being significantly better than the offline active group or both inactive groups. The existing research results show that although the drug treatment of diabetes patients can effectively control the blood sugar and other indicators, without professional guidance and daily management of diabetes patients, some diabetes patients will still have recurrent conditions and complications [21]. Our research results also indirectly reflect that online management has a stronger supervisory effect on patients. Through active or passive communication and learning, patients can continuously improve their understanding of the disease, enhance their self-management ability, and fundamentally change their unhealthy lifestyle to achieve weight loss, blood sugar control, 3B compliance, and form a virtuous cycle, ultimately achieving the goal of controlling disease progression.

Compared to blood glucose, the relationship between LDL-C and blood pressure improvement and activity level does not seem to be significant. We speculate that this may be related to the degree of changes in LDL-C and blood pressure, which are less affected by lifestyle habits such as daily diet and exercise compared to blood glucose [22]. For blood sugar, which is greatly affected by three meals a day and exercise, it is particularly important to actively participate in joint care management.

In addition to the active degree, it has a greater impact on the rate of glycated hemoglobin (HbA1c) reaching the standard, and there is an objective course of diabetes. The analysis shows that, with the prolongation of the course of diabetes, the glycated hemoglobin (HbA1c) compliance rate gradually decreases, which is similar to other research results. There are many objective factors, such as the gradual aggravation of the disease with the prolongation of the course of disease, the gradual decline of pancreatic islet function, and more complex use of drugs [23]. There are also some supervisory reasons, such as patients’ laziness in disease control and doctors’ laziness in adjusting the plan. So we should pay more attention to patients with long disease course, continuously supervise patients’ disease management through joint care, and also urge doctors to adjust their plans, resist the inertia effect brought by long disease course, achieve long-term control and management of chronic diseases, and improve disease outcomes.

## Limitations and future directions

This study has several limitations. First, it was conducted at a single center, which may limit the generalizability of the findings to other healthcare settings. Second, the lack of a control group receiving usual care precludes direct attribution of the observed improvements solely to the co-care model. Third, as noted, the cohort had a relatively high mean baseline HbA1c, suggesting the inclusion of patients with initially poorer control. This may limit the applicability of our results to all diabetic populations but highlights the model’s effectiveness in a high-need group. Future multi-center randomized controlled trials with diverse patient populations and longer follow-up are warranted.
